# Quality of life in children: the use of parent reports

**DOI:** 10.1111/dmcn.14476

**Published:** 2020-01-23

**Authors:** Hans M Koot

**Affiliations:** ^1^ Department of Clinical, Neuro‐ and Developmental Psychology Amsterdam Public Health Research Institute Vrije Universiteit Amsterdam Amsterdam the Netherlands

## Abstract

This commentary is on the original article by Boldyreva et al. on pages https://doi.org/10.1111/dmcn.14450 of this issue.

Informant variance is a perennial problem when ratings are obtained from different individuals on the same person. Low agreement between informants appears to be the rule rather than an exception. For example, in a large meta‐analytical study, Achenbach et al.^1^ found that the mean correlation between ratings of child behavioural and emotional problems from pairs of informants who played similar roles to the child (ages 1y 6mo–18y), such as pairs of parents and pairs of mental health workers, was 0.60. Between informants playing different roles the correlation was only 0.28, and between child/adolescent self‐reports and other informants’ reports the correlation was only 0.22. Findings such as these raise the question as to what to do with reports on children obtained from different informants. This becomes even more pertinent when the reports are on child/adolescent quality of life (QoL), where QoL is defined as the ‘individual’s perception of their position in life in the context of the culture and value systems in which they live and in relation to their goals, expectations, standards, and concerns’.^2^

Boldyreva et al.^3^ report that QoL, as rated by adolescents themselves, is similar in populations of young people with epilepsy or cerebral palsy (CP) when compared to adolescents in the general population. However, parent reports showed lower perceived QoL in adolescents with epilepsy or CP than in the general population, in the domains of physical well‐being, mood and emotions, autonomy, social support and peers, and social acceptance. They also report elevated levels of parental stress in the patient groups and suggested that the findings on the adolescent self‐reported QoL should serve as a source of comfort to the stressed parents.

It may be tempting to attribute parental reports of poor child QoL only to parental stress or bias. However, lack of agreement between informants may be attributable to several different sources, including situation specificity of behaviour; lack of knowledge on the part of the informant; the relationship between the child and the informant; value differences; denial, socially desirable answers, or exaggeration; and the informant’s own biases, personality, and personal problems.^4^ As yet, we do not know the best way to integrate information from different informants in a standardized way.

Indeed, parents of (chronically) ill children have an increased risk of symptoms of maladjustment (e.g. anxiety, depression) than parents of typically developing children, and these tend to influence their ratings of childhood adjustment. However, parental (especially maternal) ratings also contain substantive portions of variance (up to 50%), attributable to the child’s condition. Despite the less than optimal agreement to be expected between child and parent reports, and although parent reports of objective, easily observable aspects of child behaviour are probably more valid than parent reports on the child’s experienced QoL, parent reports cannot be discarded. First, they are indispensable when the child is too young, ill, or disabled to report reliably on their own QoL. Second, even if not highly correlated to the child’s own reports, parent reports may contain reliable and valid information that is a good indicator of the child’s condition and prospects. Comparison of child/adolescent and parent reports can be helpful when QoL ratings are used to inform treatment decisions or counselling of parent‐child pairs. For example, comparisons may reveal issues in how well the parent is informed about the child’s life; potential child or parent biases or defenses; differences in values, standards, and coping; and other sources of report discrepancies. However, little systematic knowledge is available on the backgrounds and causes of child‐parent discrepancies in reports on child/adolescent QoL. It would be helpful to initiate research addressing the backgrounds of high versus low agreement between ratings of child‐parent couples, as well as their implications for treatment and prognosis.
